# Surveillance of Antimalarial Drug-Resistance Genes in Imported *Plasmodium falciparum* Isolates From Nigeria in Henan, China, 2012–2019

**DOI:** 10.3389/fcimb.2021.644576

**Published:** 2021-04-23

**Authors:** Dongyang Zhao, Hongwei Zhang, Penghui Ji, Suhua Li, Chengyun Yang, Ying Liu, Dan Qian, Yan Deng, Hao Wang, Deling Lu, Ruimin Zhou, Yuling Zhao

**Affiliations:** Department of Parasite Disease Control and Prevention, Henan Provincial Center for Disease Control and Prevention, Henan Key Laboratory of Infectious Disease Microbiology, Zhengzhou, China

**Keywords:** Plasmodium falciparum, drug resistance, Pf*K13*, *Pfcrt*, *Pfmdr1*, *Pfdhfr*, *Pfdhps*, Nigeria

## Abstract

Malaria remains a major public health issue in Nigeria, and Nigeria is one of the main sources of imported malaria in China. Antimalarial drug resistance is a significant obstacle to the control and prevention of malaria globally. The molecular markers associated with antimalarial drug resistance can provide early warnings about the emergence of resistance. The prevalence of antimalarial drug resistant genes and mutants, including *PfK13*, *Pfcrt*, *Pfmdr1*, *Pfdhfr*, and *Pfdhps*, was evaluated among the imported *Plasmodium falciparum* isolates from Nigeria in Henan, China, from 2012 to 2019. Among the 167 imported *P. falciparum* isolates, the wild-type frequency of *PfK13*, *Pfcrt*, *Pfmdr1*, *Pfdhfr*, and *Pfdhps* was 98.7, 63.9, 34.8, 3.1, and 3.1%, respectively. The mutation of *PfK13* was rare, with just two nonsynonymous (S693F and Q613H) and two synonymous mutations (C469C and G496G) identified from four isolates. The prevalence of *Pfcrt* mutation at codon 74–76 decreased year-by-year, while the prevalence of *pfmdr1* 86Y also decreased significantly with time. The prevalence of *Pfdhfr* and *Pfdhps* mutants was high. Combined mutations of *Pfdhfr* and *Pfdhps* had a high prevalence of the quadruple mutant I_51_R_59_N_108_-G_437_ (39.0%), followed by the octal mutant I_51_R_59_N_108_-V_431_A_436_G_437_G_581_S_613_ (17.0%). These molecular findings update the known data on antimalarial drug-resistance genes and provide supplemental information for Nigeria.

## Introduction

Globally, malaria incidence and mortality have substantially reduced since 2010, and an increasing number of countries are moving toward malaria elimination. In 2016, the World Health Organization (WHO) identified 21 countries that had the potential to eliminate malaria by 2020, including China (WHO, 2018). In Henan Province, there has been no local malaria case since 2012 (Liu et al., 2014), while in 2017, no indigenous malaria cases were reported in China for the first time (Zhang et al., 2018). However, malaria remains the major public problem in sub-Saharan Africa, and Nigeria had the greatest burden of global malaria cases (27%) and malaria deaths (23%) worldwide in 2019 (WHO, 2020).

Efficacious antimalarial medicines are critical to malaria control and elimination. However, the emergence of antimalarial drug resistance increases the burden of malaria, and is one of the recurring challenges in the global fight against malaria. Monitoring antimalarial drug efficacy is necessary to provide information for treatment policies, as well as to mitigate the impact of resistance and prevent its spread. Therapeutic efficacy studies (TESs) and integrated drug efficacy surveillance (iDES) are common and reliable measures to obtain data on treatment efficacy, and the molecular markers associated with parasite resistance can provide supplemental information for TESs and iDES.

Chloroquine (CQ), as the first-line therapy for *Plasmodium falciparum* malaria, was the most common antimalarial in Africa from the 1940s to the 2000s (Nuwaha, 2001). CQ was replaced by artemisinin-based combination therapies (ACTs) in most African countries from the late 1990s to 2000s with the spread of chloroquine resistance (Flegg et al., 2013). However, CQ remains the first-line treatment for *P. vivax* in most endemic countries (WHO, 2019), while ACTs have been recommended by the WHO as the first-line treatment for uncomplicated *P. falciparum* malaria in nearly all areas, as well as for chloroquine-resistant (CQR) *P. vivax* malaria (WHO, 2015). Artemether-lumefantrine (AL), artesunate-amodiaquine (AS-AQ), and dihydroartemisinin-piperaquine (DHA-PPQ) are used as the first-line treatment for *P. falciparum* in most African countries (WHO, 2019). Sulfadoxine-pyrimethamine (SP) is recommended for the intermittent preventive treatment of malaria in pregnant women and infants (IPTp and IPTi) by the WHO (WHO, 2010).

CQR was first reported at the Thailand-Cambodia border in 1957 (Mehlotra et al., 2001; Ridley, 2002) and then confirmed in Africa in 1979 (Campbell et al., 1979). The emergence of artemisinin resistance (ART-R) in *P. falciparum* was first reported in Cambodia and later became widespread in the Greater Mekong sub-region (GMS) (Noedl et al., 2008; Dondorp et al., 2009; Yeung et al., 2009). Currently, resistance to the partner drugs of artemisinin (ART) is also common in the GMS, which affects the efficacy of ACTs. The *P. falciparum* chloroquine resistance transporter gene (*Pfcrt*) is the most reliable molecular marker of CQR, and has also been considered to be associated with resistance to ACT partner drugs, such as piperaquine (Ross et al., 2018), amodiaquine (Sá et al., 2009), lumefantrine (Sisowath et al., 2009), and mefloquine (Picot et al., 2009). The polymorphism of the Kelch 13 (*K13*) propeller domain in *P. falciparum* has been identified as a molecular marker of partial ART resistance (Ariey et al., 2014). Many non-synonymous mutations in *PfK13* have been identified (WHO, 2018); however, only a few *PfK13* mutations have been validated, and all of them have been identified in the GMS in Southeast Asia (Ménard et al., 2016). Some mutations in *PfK13* were also identified in Africa, although these have not been validated *in vivo* or *in vitro* (Taylor et al., 2014). A recent study confirmed the *de novo* emergence and clonal expansion of an ART-R *PfK13* R561H lineage in Rwanda (Uwimana et al., 2020). These findings have substantial implications for the treatment and control of malaria in Africa. *P. falciparum* multidrug resistance 1 gene (*Pfmdr1*) has been linked with the efficacy of chloroquine, mefloquine, amodiaquine, lumefantrine, artemisinin, and others (Humphreys et al., 2007; Somé et al., 2010). The polymorphisms of *Pfmdr1* at codons N86Y, Y184F, S1034C, N1042D, and D1246Y have also been shown to be linked with antimalarial drug resistance (Picot et al., 2009; Kamugisha et al., 2012). The *Pfmdr1* mutations N86Y and D1246Y, together with the *Pfcrt* mutations, can reduce the efficacy of CQ (Sá et al., 2009). The mutations of *P. falciparum* dihydrofolate reductase (*Pfdhfr*) at codons A16V, N51I, C59R, S108N/T, and I164L have been reported to be related with pyrimethamine resistance (McCollum et al., 2006). Moreover, polymorphisms of *P. falciparum* dihydropteroate synthase (*Pfdhps*) at codons S436A/F, A437G, K540E, A581G, and A613S/T are considered to be associated with sulphadoxine resistance (Vinayak et al., 2010). The quintuple mutation composed of the *pfdhfr* triple mutant 51I59R108N and the *Pfdhps* double mutant 437G540E has been reported to reduce the efficacy of SP in IPTp and IPTi (Gosling et al., 2009; Nankabirwa et al., 2010).

In Nigeria, ACTs have been recommended by the National malaria drug policy since 2005 because of the failure of CQ treatment (FMoH, 2005). However, CQ is still used to treat malaria because it is both accessible and cheap. Currently, AL and AS-AQ, as the recommended ART-based combinations, are adopted for the treatment of uncomplicated malaria in Nigeria, and the most recent TES showed that the cure rate was more than 95% (Sowunmi et al., 2017). At present, pregnant Nigerian women are also recommended to receive 3+ doses of SP to prevent malaria (NPC and ICF, 2019).

Nigeria is one of the main sources of imported malaria in China. It is important to understand the efficacy of antimalarial drugs for these imported cases. Therefore, in this study, we performed molecular surveillance of antimalarial drug-resistant genes in *P*. *falciparum* isolates imported from Nigeria in Henan Province, China, to determine their haplotypes and prevalence.

## Materials and Methods

### Sample Characteristics

All of the information on imported malaria cases in Henan Province was collected from the Disease Surveillance Information Report Management system of China Center for Disease Control and Prevention. A total of 1,522 imported malaria cases were reported in Henan Province during 2012–2019, with no indigenous cases. Nigeria is one of the main sources of imported malaria in Henan Province, and 201 of the total cases originated from here. Among these 201 cases, 167 cases were infected with *P. falciparum*. With the exception of one patient who was Nigerian working in China, the other 200 cases were Chinese people who traveled to Nigeria and returned with malaria infection. One case was female, and the others were male. The youngest was 19 years old and the oldest was 62 years old. The average age was 38.11 ± 9.188 years, and most cases were concentrated in the 21–50 years age group (86.6%, 174/201). Most of the patients were migrant workers (84.6%, 170/201).

### DNA Extraction and Amplification

The patients were diagnosed with malaria initially by blood smear microscopy and/or rapid diagnosis test (RDT) in the local hospital or County Centers for Disease Control and Prevention. The whole blood and blood smear samples of the cases were collected before antimalarial treatment and deposited in the Sample Resource Library in Henan Provincial Reference Laboratory for Malaria Diagnosis. All of the cases were confirmed to be infected with malaria parasite species using nested polymerase chain reaction (PCR) and blood smear microscopy performed at the Henan Provincial Reference Laboratory for Malaria Diagnosis (Yin et al., 2015). The genomic DNA was extracted from blood samples using QIAamp DNA Blood Mini Kits (Qiagen, USA) following the manufacturer’s instructions.

The target genes of *PfK13*, *Pfcrt*, *Pfmdr1*, *Pfdhfr*, and *Pfdhps* were amplified using nested PCR previously described (Zhou et al., 2016; Zhou et al., 2019). The primer sequences and conditions are shown in **Supplementary Material**. The amplification of the *Pfcrt* gene amplified covered the codons C72S, V73V, M74I, N75E, and K76T. The *Pfmdr1* gene contained the codons N86Y, Y184F, S1034C, N1042D, and D1246Y. The *Pfdhfr* gene was amplified to identify the polymorphism at codons A16S, N51I, C59R, S108N, and I164L. The *Pfdhps* gene covered the codons I431V, S436A, A437G, K540E, A581G, and A613S. Bidirectional sequencing of the secondary PCR products was performed by Sangon Biotech Co Ltd (Shanghai, China).

### Data Analysis

ChromasPro software v. 1.5 (https://technelysium.com.au/wp/chromaspro/) was used to assemble the forward and reverse sequences of the genes. MEGA7 (Molecular Evolutionary Genetics Analysis, https://www.megasoftware.net/show_eua) software was used to identify the mutations by comparing with their reference genomes. The reference genomes of *PfK13*, *Pfcrt*, *Pfmdr1*, *Pfdhfr*, and *Pfdhps* were from the *P. falciparum* 3D7 strain obtained from Genbank (Genbank ID: Pf3D7_1343700, Pf3D7_0709000, Pf3D7_0523000, PF3D7_0417200, and PF3D7_1324800, respectively). The data were analyzed using SSPS v.21.0 (Statistical Product and Service Solutions). The difference was compared using Chi-square or Fisher’s exact test, and a two-sided *p* value of <0.05 was considered statistically significant.

## Results

### *K13*-Propeller

The 850 bp fragments of the *K13*-propeller domain were successfully sequenced from 157 samples among 167 P*. falciparum* isolates. Two non-synonymous and two synonymous mutations were identified from four isolates: S693F, Q613H, C469C, and G496G. The total mutant prevalence was 2.5% (4/157). Two synonymous mutations were detected in 2014 and 2016, and one non-synonymous mutation was identified in 2017 and 2019.

### Pfcrt

**Figure 1:**
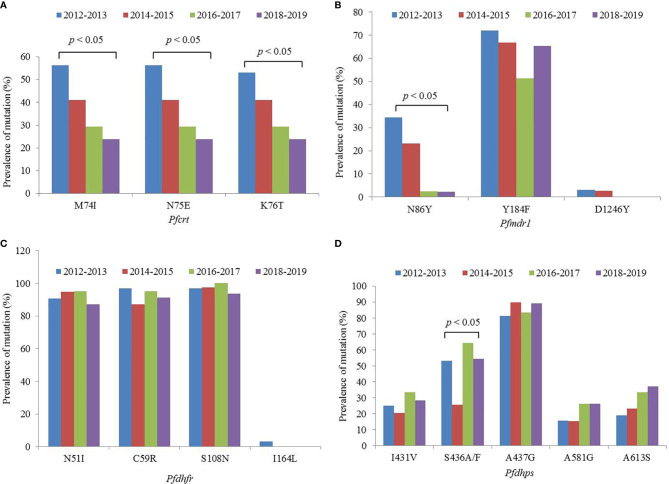
Mutant prevalence of the **(A)** *Pfcrt*, **(B)** *Pfmdr1*, **(C)** *Pfdhfr*, and **(D)** *Pfdhps* genes detected in *Plasmodium falciparum* isolates returned from Nigeria, 2012–2019.

A total of 158 out of 167 isolates were successfully sequenced on *Pfcrt*. The codons 72 and 73 were all wild type. The mutant prevalence of codons 74, 75, and 76 was 36.1% (57/158), 36.1% (57/158), and 35.4% (56/158), respectively, during 2012–2019 (**Table 1**). Four haplotypes of *Pfcrt* were identified, wild-type CVMNK (63.9%, 101/158), CVIET (32.3%, 51/158), CVIEK (0.6%, 1/158), and CV M/I N/E K/T (3.2%, 5/158) (**Table 2**). The mutant prevalence of *Pfcrt* 74I, 75E, and 76T decreased with time, and the differences showed statistical significance (74I and 75E: χ^2^ = 9.837, *p* = 0.020; 76T: χ^2^ = 8.260, *p* = 0.041) (**Figure 1A**).

**Table 1:** Mutant prevalence of the *Pfcrt*, *Pfmdr1*, *Pfdhfr*, and *Pfdhps* genes detected in *Plasmodium falciparum* isolates returned from Nigeria during 2012–2019.

Gene	SNP	Prevalence of mutation^a^ (%)								
		Total	2012	2013	2014	2015	2016	2017	2018	2019
*Pfcrt*	M74I	36.1 (57/158)	72.2(13/18)	35.7 (5/14)	42.3 (11/26)	38.5 (5/13)	20.0 (3/15)	34.6 (9/26)	26.7(4/15)	22.6(7/31)
	N75E	36.1 (57/158)	72.2(13/18)	35.7 (5/14)	42.3 (11/26)	38.5 (5/13)	20.0 (3/15)	34.6 (9/26)	26.7(4/15)	22.6(7/31)
	K76T	35.4 (56/158)	66.7(12/18)	35.7 (5/14)	42.3 (11/26)	38.5 (5/13)	20.0 (3/15)	34.6 (9/26)	26.7(4/15)	22.6(7/31)
*Pfmdr1* ^b^	N86Y	13.9 (22/158)	38.9 (7/18)	28.6 (4/14)	23.1 (6/26)	23.1 (3/13)	6.7 (1/15)	0 (0/26)	0(0/15)	3.2(1/31)
	Y184F	63.3 (100/158)	77.8 (14/18)	64.3 (9/14)	61.5 (16/26)	76.9 (10/13)	53.3 (8/15)	50 (13/26)	60.0(9/15)	67.7(21/31)
	D1246Y	1.3 (2/158)	0 (0/18)	7.1 (1/14)	3.8 (1/26)	0 (0/13)	0 (0/15)	0 (0/26)	0(0/15)	0(0/31)
*Pfdhfr* ^c^	N51I	91.8 (146/159)	100 (18/18)	78.6 (11/14)	100 (26/26)	84.6 (11/13)	86.7 (13/15)	100 (27/27)	86.7(13/15)	87.1(27/31)
	C59R	92.5 (147/159)	100 (18/18)	92.9 (13/14)	96.2 (25/26)	69.2 (9/13)	93.3 (14/15)	96.2 (26/27)	86.7(13/15)	93.5(29/31)
	S108N	96.9 (154/159)	100 (18/18)	92.9 (13/14)	100 (26/26)	92.3 (12/13)	100 (15/15)	100 (27/27)	93.3(14/15)	93.5(29/31)
	I164L	0.6 (1/159)	5.6 (1/18)	0 (0/14)	0 (0/26)	0 (0/13)	0 (0/15)	0 (0/27)	0(0/15)	0(0/31)
*Pfdhps* ^d^	I431V	27.0 (43/159)	22.2 (4/18)	28.6 (4/14)	19.2 (5/26)	23.1 (3/13)	26.7 (4/15)	37.0 (10/27)	33.3(5/15)	25.8(8/31)
	S436A	47.2 (75/159)	38.9 (7/18)	64.3 (9/14)	15.4 (4/26)	30.8 (4/13)	60 (9/15)	66.7 (18/27)	53.3(8/15)	51.6(16/31)
	S436F	2.5 (4/159)	5.6 (1/18)	0 (0/14)	7.7 (2/26)	0 (0/13)	0 (0/15)	0 (0/27)	0(0/15)	3.2(1/31)
	A437G	86.2 (137/159)	77.8 (14/18)	85.7 (12/14)	84.6 (22/26)	100 (13/13)	73.3 (11/15)	88.9 (24/27)	100(15/15)	83.9(26/31)
	A581G	21.4 (34/159)	16.7 (3/18)	14.3 (2/14)	11.5 (3/26)	23.1 (3/13)	26.7 (4/15)	25.9 (7/27)	33.3(5/15)	22.6(7/31)
	A613S	28.9 (46/159)	22.2 (4/18)	14.3 (2/14)	19.2 (5/26)	30.8 (4/13)	33.3 (5/15)	33.3 (9/27)	40.0(6/15)	35.5(11/31)

a including the mixed mutation.

b T1192L mutant was identified from one isolate in 2019.

c S120R mutant was identified from one isolate in 2015.

d E424G mutant was identified from one isolate in 2015.

**Table 2:** Haplotypes of *Pfcrt*, *Pfmdr1*, *Pfdhfr*, and *Pfdhps* genes detected in *Plasmodium falciparum* isolates returned from Nigeria during 2012–2019.

Gene (No.)	Haplotypes	No. (%)
*Pfcrt* (n=158)	Wild-type C_72_V_73_M_74_N_75_K_76_	101 (63.9)
	Double mutant haplotype CV**IE**K	1 (0.6)
	Triple mutant haplotype CV**IET**	51 (32.3)
	Mixed triple mutant haplotype CV **M/I N/E K/T**	5 (3.2)
*Pfmdr1* (n=158)	Wild-type N_86_Y_184_S_1034_N_1042_D_1246_	55 (34.8)
	Single mutant haplotype N**F**SND	80 (50.6)
	Single mutant haplotype NYSND-T1192**L**	1 (0.6)
	Double mutant haplotype **YF**SND	20 (12.7)
	Double mutant haplotype **Y**YSN**Y**	2 (1.3)
*Pfdhfr* (n=159)	Wild-type A_16_N_51_C_59_S_108_I_164_	5 (3.1)
	Single mutant haplotype ANC**N**I	1 (0.6)
	Double mutant haplotype A**I**C**N**I	5 (3.1)
	Double mutant haplotype AN**RN**I	6 (3.8)
	Double mutant haplotype ANC**N**I-S120**R**	1 (0.6)
	Triple mutant haplotype A**IRN**I	140 (88.1)
	Quadruple mutant haplotype A**IRNL**	1 (0.6)
*Pfdhps* (n=159)	Wild-type I_431_S_436_A_437_K_540_A_581_A_613_	5 (3.1)
	Single mutant haplotype IS**G**KAA	70 (40.0)
	Single mutant haplotype I**A**AKAA	11 (6.9)
	Double mutant haplotype I**AG**KAA	11 (6.9)
	Double mutant haplotype **VA**AKAA	2 (1.3)
	Double mutant haplotype I**F**AKA**S**	4 (2.5)
	Double mutant haplotype IS**G**K**G**A	2 (1.3)
	Triple mutant haplotype **VAG**KAA	8 (5.0)
	Triple mutant haplotype **V**S**G**K**G**A	1 (0.6)
	Triple mutant haplotype I**AG**KA**S**	12 (7.5)
	Triple mutant haplotype IS**G**K**G**A-E424G	1 (0.6)
	Quadruple mutant haplotype **VAG**K**G**A	2 (1.3)
	Quadruple mutant haplotype **VAG**KA**S**	2 (1.3)
	Quadruple mutant haplotype **V**S**G**K**GS**	1 (0.6)
	Quintuple mutant haplotype **VAG**K**GS**	27 (17.0)

### Pfmdr1

The fragments of *Pfmdr1* were successfully obtained from 158 isolates. No mutation was found at codons 1034 and 1042, and only two isolates had mutation at codon D1246Y. One T1192L mutant was identified for the first time in our study. The mutant prevalence of N86Y and Y184F was 13.9% (22/158) and 63.3% (100/158), respectively, during 2012–2019 (**Table 1**). Five haplotypes of *Pfmdr1* were found, including wild-type NYSND, two single mutant haplotypes N**F**SND and NYSND-T1192**L**, and two double mutant haplotypes **YF**SNLD and **Y**YSN**Y** (**Table 2**). The mutant prevalence of *Pfmdr1* 86Y decreased significantly with time (χ^2^ = 23.704, *p* = 0.000) (**Figure 1B**).

### Pfdhfr

*Pfdhfr* was successfully amplified from 159 P*. falciparum* isolates. Only five isolates were wild type, and the other 154 isolates had mutations among five codons N51I, C59R, S108N, S120R, and I164L. The mutations at codons N51I, C59R, and S108N were common, accounting for 91.8% (146/159), 92.5% (147/159), and 96.9% (154/159), respectively (**Table 1**). The *Pfdhfr* S120R and I164L mutants were identified from just one isolate each. The *Pfdhfr* S120R mutant was newly identified in this study. No *Pfdhfr* A16V mutant was found, although there was no statistical difference in the mutant prevalence among these codons during 2012–2019 (**Figure 1C**). Seven haplotypes of *Pfdhfr* were found, including wild-type, single, double, triple, and quadruple mutant haplotypes. However, the triple mutation, I_51_R_59_N_108_ haplotype, was the most common, accounting for 88.1% (140/159) (**Table 2**).

### Pfdhps

Among the 159 successfully sequenced samples, five isolates were free of mutations, and the other 154 isolates had mutations among six codons E424G, I431V, S436A/F, A437G, A581G, and A613S. There were two types of mutations at codon 436, S436A (75 isolates), and S436F (4 isolates). The mutant S436F always appeared with *Pfdhps* 613S. No *Pfdhps* K540E mutant was found. The mutation at codon A437G (86.2%, 137/159) was the most prevalent, followed by S436A/F (49.7%, 79/159), A613S (28.9%, 46/159), I431V (27.0%, 43/159), and A581G (21.4%, 34/159) (**Table 1**). Only one E424G mutant was found and was newly identified in this study. There was statistical difference about the mutant prevalence at codon S436A/F during 2012–2019 (χ^2^ = 13.152, *p* = 0.004) (**Figure 1D**). The prevalence of *Pfdhps* A613S increased yearly, but there was no significant difference (χ^2^ = 4.100, *p* = 0.251). In addition to the wild type (ISAKAA), there were 14 mutant types, including two single mutant haplotypes (ISGKAA, IAAKAA), four double mutant haplotypes (IAGKAA, VAAKAA, IFAKAS, ISGKGA), four triple mutant haplotypes (VAGKAA, VSGKGA, IAGKAS, ISGKGA-E424G), three quadruple mutant haplotypes (VAGKGA, VAGKAS, VSGKGS), and one quintuple mutant haplotype (VAGKGS). The single mutant haplotype ISGKAA (40.0%) was the most common, followed by the quintuple mutant haplotype VAGKGS (17.0%), and the triple mutant haplotype IAGKAS (7.5%) (**Table 2**).

### Combined Haplotypes of *Pfdhfr* and *Pfdhps*

Among the 167 P*. falciparum* isolates, 159 samples were successfully sequenced for *Pfdhfr* and *Pfdhps*. The results of sequencing demonstrated that only one isolate was free of mutations, and eight isolates had single-gene mutations, *Pfdhfr* or *Pfdhps*. The other 150 isolates (94.3%) had mutations in two genes simultaneously. The most frequent mutation was the quadruple mutant I_51_R_59_N_108_- G_437_, accounting for 39.0%, followed by I_51_R_59_N_108_- V_431_A_436_G_437_G_581_S_613_ (17.0%) and I_51_R_59_N_108_- A_436_ (6.9%). As no *Pfdhps* K540E mutants were detected, the combination of the *Pfdhfr* triple mutant I_51_R_59_N_108_ and the *Pfdhps* double mutant G_437_E_540_ was not observed (**Table 3**).

**Table 3:** Combination of the *Pfdhfr* and *Pfdhps* genes detected from *Plasmodium falciparum* isolates returned from Nigeria during 2012–2019.

Haplotypes	No. (%)
I_51_R_59_N_108_- V_431_A_436_G_437_G_581_S_613_	27 (17.0)
I_51_R_59_N_108_- V_431_A_436_G_437_G_581_	2 (1.3)
I_51_R_59_N_108_- V_431_A_436_G_437_S_613_	2 (1.3)
I_51_R_59_N_108_- V_431_G_437_G_581_S_613_	1 (0.6)
I_51_R_59_N_108_- V_431_A_436_G_437_	6 (3.8)
I_51_R_59_N_108_- A_436_G_437_S_613_	9 (5.7)
I_51_R_59_N_108_- V_431_G_437_G_581_	1 (0.6)
I_51_R_59_N_108_- A_436_G_437_	7 (4.4)
I_51_R_59_N_108_- F_436_S_613_	4 (2.5)
I_51_R_59_N_108_- V_431_A_436_	2 (1.3)
I_51_R_59_N_108_- G_437_G_581_	2 (1.3)
I_51_R_59_N_108_- A_436_	11 (6.9)
I_51_R_59_N_108_L_164_- G_437_	1 (0.6)
I_51_R_59_N_108_- G_437_	62 (39.0)
I_51_N_108_- G_424_G_437_G_581_	1 (0.6)
I_51_N_108_- A_436_G_437_	2 (1.3)
I_51_N_108_- G_437_	2 (1.3)
R_59_N_108_- G_437_	2 (1.3)
R_59_N_108_- A_436_G_437_	2 (1.3)
R_59_N_108_- V_431_A_436_G_437_	1 (0.6)
R_59_N_108_- A_436_G_437_S_613_	1 (0.6)
N_108_R_120_- A_436_G_437_S_613_	1 (0.6)
N_108_- G_437_	1 (0.6)
I_51_R_59_N_108_	4 (2.5)
V_431_A_436_G_437_	1 (0.6)
A_436_G_437_S_613_	1 (0.6)
G_437_	2 (1.3)
Wildtype	1 (0.6)

## Discussion

ACTs are currently considered the most effective treatment for uncomplicated falciparum malaria globally. However, the emergence and radical spread of ACT resistance represents a significant threat to malaria control and elimination. Until now, previously validated *PfK13* mutants, including F446I, N485Y, M476I, Y493H, R539T, I543T, P553L, R561H, and C580Y (the most common), have been mainly identified in Southeast Asia (WHO, 2018). Meanwhile, a few *PfK13* mutations have been reported in African *P. falciparum* isolates, including A557S, V566I, A567T, S576L, A578S, and L589I; however, none of them conferred the ART-R *in vivo* or *in vitro* (Taylor et al., 2014). The confirmation of *PfK13* R561H in Rwanda would have an important impact on the ART-R in Africa (Uwimana et al., 2020). Moreover, delayed clearance of ACTs has been reported among a few cases in Nigeria (Ajayi and Ukwaja, 2013; Wundermann and Osiki, 2017). Several mutations of *PfK13* have also been described in Nigerian isolates, including one non-synonymous mutation G665C discovered in southwestern Nigeria (Oboh et al., 2018), and six mutations (E433G, F434I, F434S, I684N, I684T, and E688K) identified in northern Nigeria, among which E433G and E688K were identified from isolates with the delayed clearance (Abubakar et al., 2020). The study performed in southwestern Nigeria in 2014 identified eight non-synonymous mutations in *PfK13*, including G496S, R539F, I543V, V566K, D584I, C580Y, and a deletion variant A557; the C580Y mutant was suspected by allelic discrimination in two samples with mixed genotypes (Tola et al., 2020). In this study, two non-synonymous mutations, S693F and Q613H, were identified in two isolates. However, none of these mutations detected in Nigerian isolates has been fully validated *in vivo* or *in vitro* for resistance to ART. Given the cases with delayed clearance to ACTs, and the fact that the *PfK13* C580Y mutation has been reported in Nigeria, urgent monitoring of the efficacy of antimalarial drugs is required to obtain an early warning signal, update the treatment policy and stop the spread of ACTs-resistance.

An increasing number of studies have shown that CQ sensitivity is recovered as a consequence of CQ withdrawal (Mwanza et al., 2016; Lu et al., 2017; Ndam et al., 2017). In Nigeria, CQ was replaced with ACTs in 2005 (FMoH, 2005). In this study, the prevalence of the *Pfcrt* mutation reduced from 72.2% in 2012 to 22.6% in 2019, and decreased steadily, and *significantly year-by-year* (**Figure 1A**). *The study of* Tola *et al.* reported that the prevalence of mutant *Pfcrt* (CVIET) was 45% in 2014 in southwestern Nigeria (Tola et al., 2020), and Lu *et al*. reported that the prevalence of *Pfcrt* 76T was 46.9% in Nigeria during 2011–2014 (Lu et al., 2017), and the prevalence of mutant *Pfcrt* was 41.9% in Nigeria during 2012–2015 in our published study (Zhou et al., 2016), the results of the three studies performed in Nigeria were similar. However, there are few recent data on the prevalence in Nigeria. In this study, the molecular monitoring of *Pfcrt* took place over an 8-year period, from 2012 to 2019, and provided sufficient information to observe the reversal of CQR in Nigeria. It is possible that the mutant prevalence of *Pfcrt* will reduce further, leading to full recovery of the sensitivity to CQ. Moreover, the dynamics of population genetics may also account for the recovered sensitivity of CQ, in that as the corresponding antimalarial drug is withdrawn, the wild type gene might segregate and increase in population.

*Pfmdr1* gene is considered to be associated with the efficacy of multiple antimalarial drugs. Meanwhile, *Pfmdr1* and *Pfcrt* are assumed to be associated with resistance to ACT partner drugs, such as amodiaquine, lumefantrine, and mefloquine (Otienoburu et al., 2019). *Pfmdr1* has also been found to be closely associated with chloroquine resistance, especially between the *Pfmdr1* N86Y and *Pfcrt* K76T (Duraisingh and Cowman, 2005), and the prevalence of *Pfmdr1* 86Y reduced with the withdrawal of chloroquine (Gupta et al., 2018). Indeed, in this study, a significant decrease in the prevalence of *Pfmdr1* 86Y was observed from 38.9% in 2012 to 3.2% in 2019. The literature review also reported that the prevalence of *Pfmdr1* 86Y reduced significantly in all of the studied countries (Otienoburu et al., 2019), which was confirmed by the current study. The prevalence of *Pfmdr1* 184F was high in this study, at 63.3% during 2012–2019. The study performed in 2007–2008 showed that the frequency of 184F was 69.0% in Nigeria (Oladipo et al., 2015). Therefore, it can be considered that the prevalence of *Pfmdr1* 184F might be maintained at a certain level for a long time in Nigeria. The prevalence of *Pfmdr1* 86Y and 184F obtained from isolates imported from Angola was 11.7 and 30.9%, respectively, during 2012–2017 (Zhou et al., 2019). In Mozambique, the frequency of *Pfmdr1* 86Y and 184F was 3.1 and 46.7%, respectively, in 2015 (Gupta et al., 2018). In other countries, the prevalence of *Pfmdr1* 86Y has been shown to be lower, whereas that of *Pfmdr1* 184F has been found to vary considerably; these observations may be related to the medication strategy of individual countries.

*Pfdhfr* and *Pfdhps* were used to monitor resistance of SP, and the level of SP resistance was considered to be related to the number of combined mutations within the two genes. The level of resistance can be divided into three groups: the quadruple mutant, *Pfdhfr* I_51_R_59_N_108_ *-Pfdhps* G_437_, is considered to be “partially resistant,” the quintuple mutant, *Pfdhfr* I_51_R_59_N_108_ *-Pfdhps* G_437_E_540_, is considered to be “fully resistant,” and the sextuple mutant, *Pfdhfr* I_51_R_59_N_108_ *-*G_437_E_540_G_581_/S_613_, is considered to be “super resistant” (Naidoo et al., 2013). In this study, *Pfdhfr* I_51_R_59_N_108_ was very common (88.1%); meanwhile, the *Pfdhps* gene had 15 haplotypes, among which the single mutant G_437_ had the highest prevalence, followed by the quintuple mutant V_431_A_436_G_437_G_581_S_613_. No *Pfdhps* K540E mutants were found. The combined mutants of the two genes showed that the quadruple mutant, *Pfdhfr* I_51_R_59_N_108_ *-Pfdhps* G_437_, was common (39.0%) and classified as “partially resistant.” Because the *Pfdhps* K540E mutant was not identified in this study, the quintuple mutant *Pfdhfr* I_51_R_59_N_108_ *-Pfdhps* G_437_E_540_ was not identified. However, 17.0% of isolates in Nigeria comprised the octal mutant *Pfdhfr* I_51_R_59_N_108_ *-Pfdhps* V_431_A_436_G_437_G_581_S_613_. Moreover, the *Pfdhps* I431V mutant was discovered recently and it has been identified in Nigeria, Cameroon, and Equatorial Guinea (Chauvin et al., 2015; Oguike et al., 2016). It has also been reported that the prevalence of *Pfdhps* 431V is 8.3, 16.7, and 6.3% in Nigeria, Cameroon, and Equatorial Guinea, respectively (Xu et al., 2019). However, the frequency of *Pfdhps* 431V reached 27.0% in this study. *Pfdhps* 431V has always been found together with other mutants, among which *Pfdhps* V_431_A_436_G_437_G_581_S_613_ was the most common. The effects of *Pfdhps* 431V on SP resistance need further study. Although no data were obtained from pregnant women or children, our findings provide supplementary information for SP resistance in Nigeria. It is necessary to monitor SP resistance continuously using the two genes to guide the IPT strategy.

There are some limitations to this study. First, the samples were passively obtained from migrants returning from Nigeria; thus, the sampling was not planned, and the sample size was not controlled. Second, the exact information about which part of Nigeria these individuals worked/lived in was unavailable. Third, although the individuals were all cured, detailed information about the treatment process and the use of antimalarial drugs was not incomplete. Fourth, the individuals were almost all Chinese people who returned from Nigeria with malaria; therefore, the prevalence of mutations among them might differ from those among native Nigerians.

This study evaluated polymorphisms and prevalence of antimalarial drug-resistance genes in imported *P. falciparum* cases from Nigeria to Henan Province, China. The mutation of *Pf K13*, associated with ACTs, was rare, and no validated mutation was found. The prevalence of *Pfcrt* and *Pfmdr1* mutants associated with the resistance of ACT partner drugs reduced gradually. Moreover, the prevalence of *Pfdhfr* and *Pfdhps* mutations was high. At present, ACTs are still effective for those returning from Nigeria with *P. falciparum* malaria infected in Henan Province. However, the validated *PfK13* R561H mutation recently observed in Rwanda has substantial implications for ART-R in Africa (Uwimana et al., 2020). The routine molecular surveillance of antimalarial drugs is more important and imperative for the imported malaria cases, especially those from Africa, and will be helpful to rationalize drug guidance for local authorities in China.

## Data Availability Statement

The raw data supporting the conclusions of this article will be made available by the authors, without undue reservation.

## Ethics Statement

The studies involving human participants were reviewed and approved by the Ethical Committee of Henan Province Center for Disease Control and Prevention. The patients/participants provided their written informed consent to participate in this study.

## Author Contributions

DZ, RZ, and YZ conceived and designed the study. HZ, SL, and CY performed the experiments. DZ was responsible for the data analysis and drafted the manuscript. SL and YL participated in the sample collection. PJ and DQ contributed to the data collection. RZ and YZ revised the manuscript. YD, HW, and DL provided the administrative coordination. All authors contributed to the article and approved the submitted version.

## Funding

The study was supported by Science and Technology Project of Henan Province (**No.** 182102310631) and Henan Provincial Medical Science and Technology Project (**No.** 2018020515, **No.** 2018020509). The funders had no role in study design, data collection and analysis, decision to publish, or preparation of the manuscript.

## Conflict of Interest

The authors declare that the research was conducted in the absence of any commercial or financial relationships that could be construed as a potential conflict of interest.
