# Supplementary material for: Surveillance of Antimalarial Drug-Resistance Genes in Imported Plasmodium falciparum Isolates From Nigeria in Henan, China, 2012–2019
Source: Front Cell Infect Microbiol. 2021 Apr 23;11:644576. doi: 10.3389/fcimb.2021.644576 (PMC8102827; doi:10.3389/fcimb.2021.644576)
Supplement: Supplementary file 1 [file Table_1.docx]

| SUPPLEMENTARY TABLE 1 Primer sequences and conditions for amplication of *Pfk13*, *Pfcrt*, *Pfmdr1*, *Pfdhfr* and *Pfshps* genes in this study | | | | |
| --- | --- | --- | --- | --- |
| Gene | Primer | Primer sequences (5'-3') | Amplification conditions | Size of PCR products (bp) |
| *PfK13* | K13-1 | CGGAGTGACCAAATCTGGGA | 95^o^C×5 min, 30 cycles of 95^o^C×30 sec, 60^o^C×90 sec, 72^o^C × 90 sec], 72^o^C ×10 min | 2097bp |
|  | K13-4 | GGGAATCTGGTGGTAACAGC |  |  |
|  | K13-2 | GCCAAGCTGCCATTCATTTG | 95^o^C×5 min, 30 cycles of 95^o^C×30 sec, 60^o^C×90 sec, 72^o^C × 90 sec], 72^o^C ×10 min | 850bp |
|  | K13-3 | GCCTTGTTGAAAGAAGCAGA |  |  |
| *Pfcrt* | Pfcrt1-F | CCGTTAATAATAAATACACGCAG | 95^o^C×5 min, 30 cycles of 95^o^C×30 sec, 56^o^C×45 sec, 72^o^C × 60 sec], 72^o^C ×10 min | 537bp |
|  | Pfcrt1-R | CGGATGTTACAAAACTATAGTTACC |  |  |
|  | Pfcrt2-F | TGTGCTCATGTGTTTAAACTT | 95^o^C×5 min, 30 cycles of 95^o^C×30 sec, 48^o^C×45 sec, 72^o^C × 60 sec], 72^o^C ×10 min | 145bp |
|  | Pfcrt2-R | CAAAACTATAGTTACCAATTTTG |  |  |
| *Pfmdr1* | Pfmdr86 1F | TTAAATGTTTACCTGCACAACATAGAAAATT | 95^o^C×5 min, 30 cycles of 95^o^C×30 sec, 50^o^C×45 sec, 72^o^C × 60 sec], 72^o^C ×10 min | 612bp |
|  | Pfmdr86 1R | CTCCACAATAACTTGCAACAGTTCTTA |  |  |
|  | Pfmdr86 2F | TGTATGTGCTGTATTATCAGGA | 95^o^C×5 min, 30 cycles of 95^o^C×30 sec, 50^o^C×45 sec, 72^o^C × 60 sec], 72^o^C ×10 min | 526bp |
|  | Pfmdr86 2R | CTCTTCTATAATGGACATGGTA |  |  |
|  | Pfmdr1246 1F | AATTTGATAGAAAAAGCTATTGATTATAA | 95^o^C×5 min, 30 cycles of 95^o^C×30 sec, 50^o^C×45 sec, 72^o^C × 60 sec], 72^o^C ×10 min | 880bp |
|  | Pfmdr1246 1R | TATTTGGTAATGATTCGATAAATTCATC |  |  |
|  | Pfmdr1246 2F | GAATTATTGTAAATGCAGCTTTA | 95^o^C×5 min, 30 cycles of 95^o^C×30 sec, 50^o^C×45 sec, 72^o^C × 60 sec], 72^o^C ×10 min | 799bp |
|  | Pfmdr1246 2R | GCAGCAAACTTACTAACACG |  |  |
| *Pfdhfr* | Pfdhfr1-F | TTTATGATGGAACAAGTCTGC | 95^o^C×5 min, 30 cycles of 95^o^C×30 sec, 52^o^C×45 sec, 72^o^C × 60 sec], 72^o^C ×10 min | 650bp |
|  | Pfdhfr1-R | CTAGTATATACATCGCTAACA |  |  |
|  | Pfdhfr2-F | TGATGGAACAAGTCTGCGACGTT | 95^o^C×5 min, 30 cycles of 95^o^C×30 sec, 46^o^C×45 sec, 72^o^C × 60 sec], 72^o^C ×10 min | 594bp |
|  | Pfdhfr2-R | CTGGAAAAAATACATCACATTCATATG |  |  |
| *Pfdhps* | Pfdhps1-F | GATTCTTTTTCAGATGGAGG | 95^o^C×5 min, 30 cycles of 95^o^C×30 sec, 51^o^C×45 sec, 72^o^C × 60 sec], 72^o^C ×10 min | 770bp |
|  | Pfdhps1-R | TTCCTCATGTAATTCATCTGA |  |  |
|  | Pfdhps2-F | AACCTAAACGTGCTGTTCAA | 95^o^C×5 min, 30 cycles of 95^o^C×30 sec, 51^o^C×45 sec, 72^o^C × 60 sec], 72^o^C ×10 min | 711bp |
|  | Pfdhps2-R | AATTGTGTGATTTGTCCACAA |  |  |
